# Notes and News

**Published:** 1891-02-07

**Authors:** 


### Notes and News.

Collected and Communicated.

Secretaries and others who wish our representative to attend their meetings are invited to give us at least a week's notice. Failing this, it is not possible to make satisfactory arrangements, and to avoid disappointment we have thought it well to give this caution.

The first of the three dances in aid of the North-West London Hospital took place in the large suite of the Portman Rooms on January the 21st, and was a very great success. The rooms were artistically decorated with palms, arums, and daffodils, and there were 360 guests present. The Hungarian Band played in splendid style. Among the people present we noticed Lady Galsworthy, Mrs. Cecil Lee, Mrs. Blandford, Mrs. MacTear, Mrs. Durham, Mrs. Fearon, Mrs. George Richardson, Mrs. Bucknall, Mrs. Hare, Mrs. Henry Wilson, Mrs. Sich, Mrs. Routledge, Mrs. Lee, Mrs. Stowers, and Mrs. Tuke. Mrs. George Herring and the Hon. Secretary, Dr. Walter Sibley, were well pleased with the success of their undertaking, and hope to hand over a large cheque for the funds of the hospital, which are not in a very satisfactory condition. The other two dances are on February the 3rd and 16th.

The annual meeting of the Governors of the Metropolitan Hospital, which has a separate ward for patients of the Jewish persuasion, was held on Wednesday last at the institution in Kingsland Road. Mr. Joseph Fry (chairman of the committee) presided, and among those present were Mr. J. R. Pike, Mr. C. J. Thomas, Mr. Coleman Defries, Mr. Henry Lovegrove, Dr. H. J. Fotherby, Dr. A. H. Brewer, Dr. J. W. Hunt, and Dr. B. J. Morison, Lieut.-Colonel Montefiore, the Rev. Septimus Buss, and the Rev. R. S. Hassard. The Secretary (Mr. C. H. Byers) read the fifty-fifth annual report of the Committee, which stated that the in-patients had increased from 489 in 1889 to 709 last year, and the new out-patient cases from 9,482, representing 16,411 attendances, to 14,104, representing 23,074 attendances. In the provident department there had been 43,208 attendances, making the total in both departments 66,506, as against 59,129 in the previous year. The receipts for the year had amounted to £6,132, and the expenditure to £7,528. To close up all the accounts connected with the building of the present hospital, the Committee had been obliged to borrow £6,000 on mortgage, the interest of which, at four per cent., amounted to £240 per annum. If that expenditure could be obviated, the Committee would be able to open at least five more beds, and with that object in view they intended shortly to make an earnest appeal to all friends of the hospital to help pay off the mortgage. On the motion of the Chairman, seconded by Mr. Pike, the report was adopted.

The annual general meeting of the Governors and friends of the Norwich Jenny Lind Infirmary was held at the institution in Pottergate Street. The Lord Bishop presided, and there was a large attendance, including several ladies. The Secretary read the report of the Committee of Management, from which it was evident that increased accommodation in the hospital is much wanted, the medical staff having frequently to complain of the wards being over-crowded. The Committee consider that the enlargement of the Infirmary should be no longer delayed; the only question was whether the present fabric should be enlarged, or whether a new hospital should be built on a new site. The cost of alteration and repairs to the present building has amounted to nearly £600 in the last five years, and it is still far behind in the structural requirements of a modern hospital. The site also is too limited. They have, therefore, come to the conclusion the best policy will be to erect a new building on as high and open a site as can be procured. Financially the affairs of the hospital are satisfactory; they have a balance in hand of £257 18s.

At the annual meeting of the Governors of the Lincoln County Hospital, the Rev. H. W. Hutton presided. The Secretary read an unusually satisfactory report, from which it appeared that the income of the hospital had been sufficient not only to meet all expenses, and to wipe off a debt of £66 4s. 1d. which was owing at the beginning of the year, but to enable them to start the new year with a balance of £285 8s. 1d. in their favour. It was mainly, Mr. Danby said, to the exertions put forward in raising collections on Hospital Sunday and Hospital Saturday that they owed their present satisfactory condition; and that if there was the slightest relaxation or falling off in either of those directions, there would soon again be a balance on the wrong side. The accounts were passed. The Chairman then rose to announce that he had received a letter from Mrs. Wilkinson, stating that her husband wished to withdraw his application to be made consulting surgeon to the hospital. The Rev. Sub-dean Clements proposed the following resolution: That this Board hears with great regret of the resignation of Mr. T. M. Wilkinson, who for thirteen years has fulfilled his duties as surgeon most assiduously and successfully, and whose courtesy and kindness of demeanour to all its inmates will make his loss to the hospital much felt."

The Quarterly Court of the Governors of Addenbrooke's Hospital, Cambridge, was held at the hospital lately. The Rev. Dr. Campion presided. After the Secretary had read his report, which was of a very satisfactory nature, an interesting discussion on the subject of out-patients took place. The Rev. Henry Hall stated that these patients had increased very greatly in number during the last five years, and he was of opinion that the developing of the provident medical dispensary would be advantageous both to the hospital and the patients. Dr. MacAlister agreed with Mr. Hall, and added that he had noticed, not only a large increase in the number of patients, but he had been struck with their general appearance. They were often very well dressed, and he had known patients drive up in their own traps. He had called the attention of some of the governors to the fact that such patients were not in need of charity. Dr. Humphry stated that his experience of surgical out-patients was, that in spite of their respectable appearance, they were frequently found to be in very pressing circumstances. The greater number were certainly poor people, and came struggling long distances to get the help they got, and which they highly appreciate. Dr. MacAlister said he thought it was almost impossible for the medical officers to act as detectives in the matter on account of the numbers who were waiting to be attended to. No decision on the subject was arrived at, and the meeting adjourned.

